# Evaluation of Hydroxyethyl Cellulose Grades as the Main Matrix Former to Produce 3D-Printed Controlled-Release Dosage Forms

**DOI:** 10.3390/pharmaceutics14102103

**Published:** 2022-10-01

**Authors:** David Hartzke, Axel Pössl, Peggy Schlupp, Frank E. Runkel

**Affiliations:** 1 Department of Life Science Engineering, Institute of Bioprocess Engineering and Pharmaceutical Technology, Technische Hochschule Mittelhessen—University of Applied Sciences, Wiesenstrasse 14, 35390 Giessen, Germany; 2 Department of Biology and Chemistry, Justus Liebig University, Ludwigstrasse 23, 35390 Giessen, Germany; 3 Department of Pharmaceutics and Biopharmaceutics, Philipps University Marburg, Robert-Koch-Strasse 4, 35037 Marburg, Germany

**Keywords:** 3D printing, fused deposition modeling, hot-melt extrusion, cellulose ether, dissolution, drug delivery system, controlled release

## Abstract

Diclofenac sodium tablets were successfully prepared via hot-melt extrusion (HME) and fused deposition modeling (FDM), using different molecular-weight (Mw) grades of hydroxyethyl cellulose (HEC) as the main excipient. Hydroxypropyl cellulose (HPC) was added to facilitate HME and to produce drug-loaded, uniform filaments. The effect of the HEC grades (90–1000 kDa) on the processability of HME and FDM was assessed. Mechanical properties of the filaments were evaluated using the three-point bend (3PB) test. Breaking stress and distance were set in relation to the filament feedability to identify printer-specific thresholds that enable proper feeding. The study demonstrated that despite the HEC grade used, all formulations were at least printable. However, only the HEC L formulation was feedable, showing the highest breaking stress (29.40 ± 1.52 MPa) and distance (1.54 ± 0.08 mm). Tablet drug release showed that the release was Mw dependent up to a certain HEC Mw limit (720 kDa). Overall, the release was driven by anomalous transport due to drug diffusion and polymer erosion. The results indicate that despite being underused in FDM, HEC is a suitable main excipient for 3D-printed dosage forms. More research on underutilized polymers in FDM should be encouraged to increase the limited availability.

## 1. Introduction

There is growing interest in the use of 3D printing as a manufacturing tool for drug delivery systems with unique properties for individualized therapy [1], such as tailored drug dissolution profiles [2] or multi-component dosage forms [3]. Over the last decade, several researchers successfully broadened the knowledge and usability of pharmaceutical 3D printing. They investigated the use of multiple drugs, different excipient additives, and the manufacturing process itself, to gain a better understanding for a pharmaceutical applicability [4,5].

Today, there are several 3D-printing techniques available, especially for pharmaceutical use [5]. The most common 3D-printing technique for fused deposition modeling (FDM) requires a drug-loaded filament as a feedstock. The filament mostly consists of thermoplastic polymers mixed with additional additives, including plasticizers, lubricants, or fillers, as well as excipients and the active pharmaceutical ingredient (API) [6]. During the printing process, the filament is fed and melted in a heated print head and the melt is deposited precisely through a small heated nozzle onto a building plate, in order to construct a computer-designed model layer by layer [2]. This technique enables the fabrication of dosage forms with highly accurate internal and external geometries and API distribution, which, in turn, allows for tailoring the exact drug release, offering opportunities for an individualized therapy [7]. 

The required filaments can be produced by hot-melt extrusion (HME), a widely used pharmaceutical manufacturing method [8]. During HME, the raw materials are melted and mixed by one or two rotating screws [7,9] as they are conveyed through a heated barrel to the extrusion die to form a continuous filament strand. For the preparation of filaments in the pharmaceutical field, twin-screw extruders are preferred over single-screw extruders due to the superior mixing capability [10]. HME is often used to produce molecular dispersions of poorly water-soluble APIs in polymeric matrices to improve their solubility [11]. It can further be used for taste masking [12,13] and the manufacturing of drug delivery systems with different release profiles [14,15,16]. 

In this context, different pharmaceutical-grade polymers have been investigated as excipients for HME, followed by FDM, which enables the extrusion of filaments that can be printed as immediate or modified release dosage forms [4]. Since oral dosage forms are the most commonly used route of administration, a recent study [17] investigated the role and use of polymers in additive manufacturing of solid oral dosage forms and identified about 70 potentially suitable pharmaceutical-grade polymers. However, only about 30 of them are currently used in various additive manufacturing techniques, with some of these polymers, such as polylactic acid or polyvinyl alcohol, being more frequently utilized compared to others [6,17]. Unfortunately, the available pharmaceutical-grade polymers often result in filaments that have insufficient mechanical properties [9], leading to different printing defects. While filaments that are too soft tend to deform between the feeding gears, brittle filaments break under the force of the gears or inside the bowden tube [17]. In addition, the polymers intended for FDM have to possess suitable thermal properties, including thermoplasticity [17]. Ideally, the polymers should also have a favorable processing window where glass transition temperature (*T_g_*) or melting temperature (*T_m_*) tend to be low, while the degradation temperature (*T_d_*) is preferably high to avoid degradation and allow for a sufficient range to optimize the printing results. However, there are many polymers, such as cellulose, xanthan, or starch, that do not possess these favorable thermal and mechanical properties [17] but can still offer advantages for the printed form when combined with favorable excipients or additives for 3D printing [18,19]. Since the number of polymers available for 3D printing is limited [9,20,21,22] and needs to be expanded to provide new opportunities for formulation development and potential personalization of therapies, further investigations on the usability of unused polymers are necessary, for example, by combining them with beneficial excipients or additives. 

In addition to the frequently used polyethylene oxide, polyvinyl alcohol, or polyvinylpyrrolidone [17], cellulose ethers, such as hydroxypropyl cellulose (HPC) or hydroxypropyl methylcellulose, which serve as hydrophilic matrices [23], are often selected for 3D printing [4,6]. HPC, a non-ionic, semi-crystalline polymer with a low glass transition temperature and good plasticity, allows for processing at relatively low temperatures [24,25]. Depending on its molecular weight (Mw), the drug release differs, which makes its use appropriate for a controlled drug release [4,26]. Due to acceptable mechanical properties, HPC has also been processed without the need for further additives [27]. Additionally, low-Mw HPC grades have been shown to produce chemically stable formulations with poorly and highly water-soluble APIs after being hot-melt mixed [26], making HPC a versatile excipient, suitable for HME and the 3D printing of different drug delivery systems. 

Another cellulose ether that is often used in the pharmaceutical and cosmetics industry, but not yet in 3D printing, is hydroxyethyl cellulose (HEC). HEC is a non-ionic and water-soluble polymer, mostly used as a thickening and gelling agent or coating material [28]. Due to its wide range of available Mw grades, which offers a potential to tailor drug release, HEC seems to be a promising excipient for drug delivery systems with controlled release profiles. Nevertheless, its thermoplastic characteristics are poor compared to other cellulose ethers, such as HPC [29], which might explain the lack of available studies for HEC in thermal manufacturing processes, such as FDM. To our best knowledge, HEC has only been used in a few studies where pharmaceutically driven HME or FDM were involved. Here, it was used as a suspending agent in 3D-printed dosage forms (5%*w/w* [30]) or as a release modifier in hot-melt extrudates (up to 36%*w/w* [31]) in relatively small amounts. For a further evaluation of the usability of HEC in FDM and to potentially increase the current number of available polymers, more investigations need to be conducted. 

Therefore, we aimed to develop filaments with HEC as the main matrix former to produce dosage forms loaded with the BCS-II class active ingredient diclofenac sodium and to evaluate both the influence of HEC on filament extrusion and 3D printing in particular, along with the drug release properties. In this context, we would like to point out that the development of a gastro-resistant shell filament was not within the scope of our work. However, if the dosage form targets the small and large intestine, respectively, for drug release, a gastro-resistant shell is inevitable [32]. For the assessment of four different HEC grades (Mw from 90 kDa to 1000 kDa), we investigated the thermal behavior and solid-state with commonly used thermogravimetric analysis (TGA), differential scanning calorimetry (DSC), and x-ray diffraction (XRD). Furthermore, we determined mechanical properties (breaking stress and breaking distance) of the filaments and conducted 3D-printing experiments to determine feedability, printability, and printing parameters for the different formulations. Finally, the resulting dosage forms were further analyzed in terms of their drug release profile and mechanism.

## 2. Materials and Methods

### 2.1. Materials 

We tested the hydroxyethyl cellulose grades Natrosol™ 250 L pharm (90 kDa), G pharm (300 kDa), M pharm (720 kDa), and HX pharm (1000 kDa), kindly provided by Ashland Industries Deutschland GmbH, Düsseldorf, Germany, and hydroxypropyl cellulose SSL (40 kDa), kindly provided by Nisso Chemical Europe GmbH, Düsseldorf, Germany. Diclofenac sodium (Ph.Eur. grade) was purchased from Caesar & Loretz GmbH, Hilden, Germany. Potassium dihydrogen phosphate and disodium hydrogen phosphate (Ph.Eur. grade, Carl Roth GmbH, Karlsruhe, Germany) were used for buffer preparation.

### 2.2. HME for Filament Production and Diameter Determination

Prior to HME, the polymers were dried at 60 °C for 24 h. Formulations (Table 1, total mass 100 g) were then pre-mixed using a mortar and pestle to avoid powder agglomerates. The resulting blends were transferred to a ZD 12 FB-C-1M-200/100 gravimetrical dosage unit (Three-Tec GmbH, Seon, Switzerland) and the feed rate was adjusted according to the used extrusion screw speed to allow continuous feeding. HME was carried out using the ZE 12 mm co-rotating twin-screw extruder, length/diameter ratio = 40:1 (Three-Tec GmbH, Seon, Switzerland), with conveying elements. Screw speed was adjusted individually to achieve uniform filament diameters (±0.05 mm). The screw torque (upper limit = 15 Nm) and barrel temperatures were monitored continuously. The temperatures of the six heating zones were set individually for each formulation to allow for a continuous filament production. The heating zone next to the feeding zone was at least 20 °C cooler to avoid clogging. The material was passed through a 2.85 mm extrusion round die and cooled on a conveyer belt (Three-Tec GmbH, Seon, Switzerland) and the resulting filament diameter was measured using an inline IG-028-CCD-laser-micrometer (Keyence Deutschland GmbH, Neu-Isenburg, Germany). The filament was cooled to room temperature and stored in a sealed plastic bag in the dark for further analysis.

### 2.3. 3D Printing for Tablet Production

3D printing was performed one day after filament production using the Ultimaker S5 (Ultimaker B.V., Utrecht, The Netherlands). The print head used was suitable for 2.85 mm filaments and was equipped with an AA 0.4- or BB 0.8 mm nozzle. The tablets were designed as a cylindrically shaped object with a diameter of 10 mm and height of 5 mm, using the open-source tool Autodesk^®^ Tinkercad^®^ (Autodesk Inc., San Rafael, CA, USA). Cura (v4.4 and newer, Ultimaker B.V., Utrecht, The Netherlands) was used for the slicing and printing of the tablets. If not otherwise indicated, all printing parameters for the tablets were based on the Cura printing preset for PLA. The glass print bed was heated individually for each formulation to achieve a good adhesion. The tablet infill for all formulations was 100% with lines as an infill pattern. The tablets had two fully printed layers at the top and the bottom and two outer-wall lines. In the cases where filament feedability was not given due to breakage of the developed filament, commercial PLA filament was used as a “feeding” filament, serving as a piston to push short filaments into the print head. Each formulation was printed at least 10 times to assure filament printability. 

### 2.4. Thermogravimetric Analysis of Raw Excipients

Raw excipients were analyzed by thermogravimetric analysis on a TG 209 *F1 Iris* device (NETZSCH-Gerätebau GmbH, Selb, Germany) to determine the degradation temperature (*T_d_*). Samples of about 10 mg were analyzed in an open aluminum oxide pan (Mettler-Toledo GmbH, Gießen, Germany) with a heating rate of 20 K·min^−1^ and a nitrogen flow rate of 20 mL·min^−1^. Excipients were scanned from room temperature to at least 400 °C. The data were analyzed using NETZSCH *Proteus* v6.1.0 (NETZSCH-Gerätebau GmbH, Selb, Germany). Degradation temperatures were determined using the extrapolated onset temperature according to the ISO 11358-1 standard [33]. The raw data were plotted using OriginPro v2021b (OriginLab Corporation, Northampton, MA, USA).

### 2.5. Differential Scanning Calorimetry 

Raw excipients and tablets were analyzed via DSC (DSC 1 Star System DSC/700/304, Mettler-Toledo GmbH, Gießen, Germany) to confirm a solid state of the drug. Therefore, samples of 10 to 15 mg were analyzed in a sealed aluminum pan (Mettler-Toledo GmbH, Gießen, Germany) with a punctured lid. The heating rate for the DSC measurement was 20 K·min^−1^, starting at 30 °C until reaching 300 °C and vice versa for the cooling cycle. The nitrogen flow rate was 40 mL·min^−1^. To erase the thermal history of raw excipients, the DSC measurements of physical mixtures were carried out in two heating/cooling cycles. The first heating cycle was performed until 120 °C to allow for the evaporation of water. Only the second heating cycle was chosen for analysis. The raw data were plotted using OriginPro v2021b (OriginLab Corporation, Northampton, MA, USA).

### 2.6. X-ray Diffraction 

XRD measurements were conducted to identify the solid state of the raw excipients and tablets. Here, 3D-printed dosage forms were investigated as printed round discs (25 mm diameter and 1 mm height). Therefore, the samples were analyzed using a D2 Phaser (Bruker Corporation Billerica, MA, USA), which was equipped with a copper anode (1.54184 Å) at 30 kV and 10 mA to generate X-rays and an SSD 160-2 detector in 1D mode using a full opening of 5.80°. The scanning range was 5–50° 2-Theta with a step size of 0.02° and 1 s measurement time per step. The samples were prepared in a zero background PMMA sample holder. The raw data were plotted using OriginPro v2021b (OriginLab Corporation, Northampton, MA, USA).

### 2.7. Mechanical Analysis: Three-Point-Bend Test

A three-point-bend (3PB) test was conducted to determine the influence of HEC on mechanical properties of the extruded filaments. Mechanical properties were tested on a texture analyzer (inspekt table blue, Hegewald & Peschke Mess- und Prüftechnik GmbH, Nossen, Germany), using a three-point bending equipment with two supports and one loading pin. Filaments were cut in 50 mm pieces and the diameter was measured using the CCD-laser-micrometer. The filaments were placed on the support pins with a gap of L = 30 mm. Measurements were taken using a test speed of 10 mm·min^−1^. Testing was stopped after sample fracture. Five replicates of each filament were tested. The data were collected using LabMaster software (v. 2.3.5.9) and analyzed using OriginPro v2021b (OriginLab Corporation, Northampton, MA, USA). The breaking distance (mm) and the maximum force applied (N) were measured. Breaking stress σ (N·mm^−^²) (1) was calculated using Equation (1) [34].

(1) $$\sigma_{f}=\frac{8\cdot F\cdot L}{\pi \cdot d^{3}}\;\left(circular\;cross\;section\right)$$

where

$\sigma_{f}$ is the stress in N·mm^−^².
*F* is the applied force in N.
*L* is the support pin gap in mm.
*d* is the diameter of the filament in mm.

### 2.8. Drug Content, Drug Dissolution, and Drug Release Mechanism

The drug content of the filaments following extrusion was determined (*n* = 3). Therefore, filaments were gently crushed and fully dissolved within 2 h in a stirred phosphate buffer (pH 6.8) at room temperature. The drug content was analyzed at 276 nm by UV-Vis spectroscopy, using the Cary 60 UV-Vis Spectrophotometer (Agilent Technologies Deutschland GmbH, Waldbronn, Germany). 

The drug release profiles of the tablets were determined using 708-DS dissolution apparatus (Agilent Technologies Deutschland GmbH, Waldbronn, Germany) at 37.0 ± 0.5 °C and 100 rpm in 200 mL phosphate buffer (pH 6.8) according to Ph.Eur. Dissolution Apparatus 2. Samples of 1.5 mL were automatically drawn and replaced with preheated buffer at 12 time points over a period of 24 h, using an 850-DS dissolution sampling station (Agilent Technologies Deutschland GmbH, Waldbronn, Germany). The samples (*n* = 3) were then analyzed by UV-Vis. 

To determine the drug release mechanism, the drug release data (mean ± standard deviation, *n* = 3) were plotted and different mathematical models (Table 2, Equations (2)–(5)) were fitted using the DDSolver Microsoft^®^ Excel Add-in developed by Zhang et al. [35]. As suggested for the used Korsmeyer-Peppas model [36], only the first 60% of released drug was evaluated. The best fitting model was defined, based on the highest adjusted coefficient of determination (adj. R²) [37].

## 3. Results and Discussion

### 3.1. Thermal Stability of Raw Materials and Evaluation of Extruded Filaments

**Figure 1:**
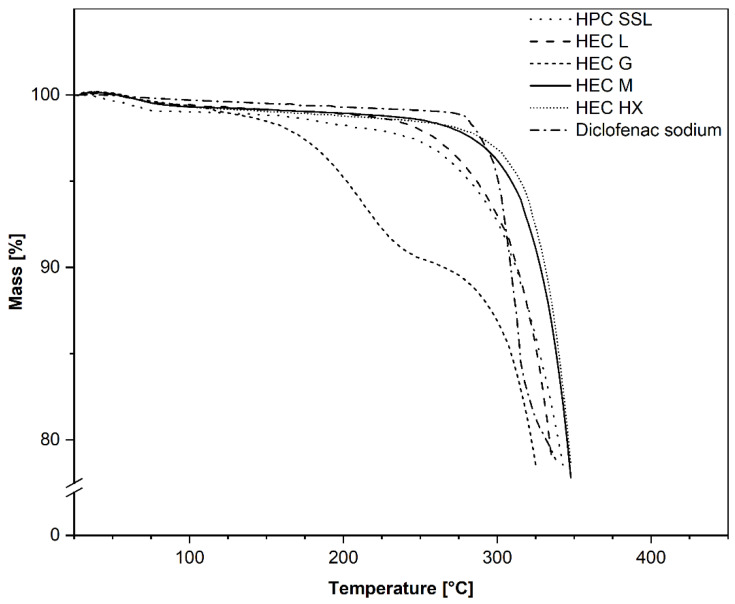
Thermogravimetric analysis of different raw excipients, using a TG 209 F1 Iris device with 20 K·min^−1^ heating rate and 20 mL·min^−1^ nitrogen flow.

Prior to extrusion, the degradation temperature of the raw excipients was determined to identify the upper limit for thermal processes. TGA measurements showed that diclofenac sodium degradation started at *T_d_* = 298 °C. The results further revealed that the first indication of degradation of the polymers started at 250 °C (Figure 1), as previously reported for HEC [38], with the highest rate of degradation at around 300 °C. The first mass loss step (~10%) of HEC G is attributed to water evaporation. Consequently, HME and 3D printing should be possible at 200 °C without any degradation of the drug or polymers. 

Initial extrusion tests of the various HEC grades without additional excipients revealed difficulties. All grades showed operating torque values near or at a maximum, even at very low screw speed and elevated temperatures (Supplementary Data: Table S1). Moreover, the filaments showed rough surfaces with extrusion defects, especially for HEC HX. This resulted in irregular filament diameters and a high brittleness. As a result, further optimization was carried out, using HPC SSL as an additional excipient due to its favorable extrudability. 

Optimized formulations (Table 3) showed a good extrudability, resulting in filaments with only little deviations in filament diameter (<0.05 mm). Continuous HME was possible at temperatures as low as 135 °C for all formulations, indicating their suitability for hot-melt extrudates of thermosensitive APIs. In accordance with this, Sanoufi et al. [31] reported that a proper extrusion of formulations containing HEC L (up to 36%*w/w*) is possible at 140 °C. Depending on the HEC grade used, differences in maximum torque and, therefore, screw speed, were observed. HEC with high Mw (in F-M and F-HX) resulted in higher extruder torque values compared to F-L and F-G, which led to a reduction in screw speed to allow good extrusion. This is attributed to the differences in melt viscosity, which is generally considered to be Mw dependent. In particular, higher Mw causes a higher melt viscosity due to polymer chain entanglement [39]. In addition to a more difficult process due to the poorer thermoplastic properties of HEC compared to others [29], the extrusion results indicate the possibility to make underutilized polymers usable by optimizing formulations through the addition of suitable excipients.

### 3.2. Mechanical Stability, Feedability, and 3D-Printing Assessment of HEC-Based Filaments

**Figure 2:**
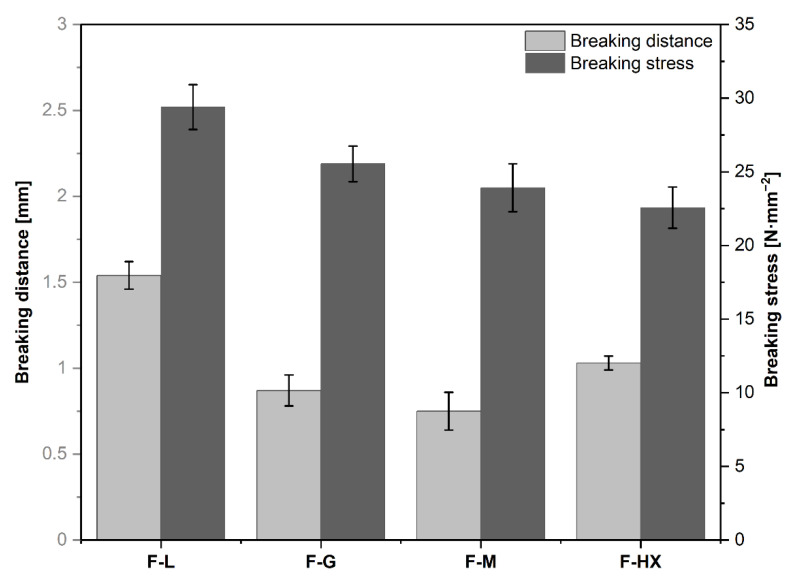
Mechanical properties of extruded filaments. Breaking distance and breaking stress were determined in a three-point-bend test (*n* = 5). Data are presented as mean ± standard deviation.

Printability is basically understood as a combination of three crucial steps, namely filament feeding, melt deposition, and adhesion [17]. For the scope of this manuscript, we took the filament feeding step out of the general definition and referred to it as “feedability” throughout the manuscript, as we associate the investigated mechanical properties (Figure 2) of the filaments to this step. Consequently, a filament designated as feedable (Table 4) was able to withstand the forces applied during the feeding step to be properly fed into the printhead. If a filament was not feedable, a supportive PLA “feeding” filament was used as a piston to push the filament into the printhead to enable printing. We, therefore, defined the term “printability” as the ability of the filaments to be reproducibly printed into the desired dosage form based on the underlying 3D model, with or without a PLA “feeding” filament. 

To assess mechanical properties, the produced filaments were investigated regarding their stiffness and brittleness, using a 3PB test. Stiffness and brittleness of filaments are regarded as the most essential parameters for successful 3D printing [9,40]. Only the formulation F-L was properly fed into the print head without a rupture of the filament (Table 4). In all other cases, the filaments were, in fact, effectively conveyed by the feeding gears but broke inside the Bowden tube as soon as the printer started moving. This indicates that the use of a direct-drive extruder could be beneficial for these filaments and could be investigated in future studies. 

In connection with the mechanical stability studies (Figure 2), it was remarkable that the feedable F-L filaments possessed the highest breaking stress (29.40 ± 1.52 MPa) and breaking distance (1.54 ± 0.08 mm) of all four formulations. This indicates, and has been argued by other researchers [41], that there is a certain threshold that filaments need to exceed in order to be feedable. Several publications have described a correlation between the mechanical properties and feedability of the filaments [9,34,40], so Quodbach et al. [42] reviewed mechanical results and postulated that for filaments, a breaking distance > 1.0–1.5 mm and a breaking stress > 28.82–30.63 MPa, obtained by a 3PB test, are advantageous for proper feeding and printing in specific printers. Both criteria were met by the F-L formulation, which was the only feedable formulation to be fed into the Ultimaker S5. All other formulations had lower values and were not feedable, consistent with the described thresholds. Unexpectedly, F-HX shows a slight increase in breaking distance compared to F-G and F-M, although it has a higher Mw. In addition to Mw, the mechanical properties of polymers are influenced by several factors, such as the degree of crosslinking, crystallinity, molecular weight distribution, hygroscopy, or even ambient temperature [43,44]. Since the mechanical measurements were all performed on the same day, the external influencing factors, such as ambient temperature, should be kept as low as possible; nevertheless, it cannot be completely excluded. The same applies to the hygroscopic properties of our excipients [45]. The filaments were stored in a sealed plastic bag in the dark to minimize environmental impact, but the storage time prior to the mechanical measurements was between 1 and 4 days. Since water can act as a plasticizer [43], this could also affect the mechanical properties. We also assume that intermolecular crosslinking or molecular weight distribution could be responsible for the observations made. To confirm this assumption, however, further investigation is necessary.

Many different test conditions for a mechanical analysis of filaments have been applied so far [9,40,46]. The results of these mechanical tests were discussed in each case, with printing experiments on specific 3D printers. Due to the individuality of the different printers, the results obtained regarding a prediction of feedability of the filaments based on the mechanical properties can only serve as an orientation for other printers and should be evaluated individually for each new printer setup. This is mainly due to the different printer configurations available, e.g., Bowden tube or direct drive in FDM printers, where the filaments need to exhibit different mechanical properties in order to be fed properly. For example, FDM printers with a Bowden tube can hardly be used with flexible filaments because the filament will deform when pushed inside the Bowden [47]. However, even if these filaments cannot be fed in printers with Bowden tubes, feeding in printers with direct drive may be possible due to the shorter distance between feed gears and nozzle [47]. 

Although only the F-L filament was feedable without an additional feeding support, all developed formulations were printable (Table 4). While the 0.4 mm nozzle resulted in constant clogging, which could be caused by the high viscosity of the melt [40], the 0.8 mm nozzle allowed good 3D printing. The drug-loaded tablets were printed at temperatures between 195 °C (F-G, F-M, F-HX) and 200 °C (F-L), which is within the range for HPC 3D printing [4] and close to the temperature described in a study using 5 wt% HEC H as a formulation ingredient [30]. However, a further increase in temperature led to no visible improvement in printability, whereas lowering the temperature resulted in an inconsistent melt flow and a print failure. 

### 3.3. Characterization of Solid Dosage Forms 

The printing results (Table 5) of the oral dosage forms revealed a low variability in tablet weight (≤4%) among the four formulations, indicating a reproducible printing process. The specified tablet dimensions of the STL model were 10 mm in diameter and 5 mm in height. The observed larger tablet diameter could be caused by differences in the melt flow of the developed filament, compared to the PLA preset selected in the Slicer software. With the selection of the PLA preset, the software adjusted all required values (e.g., extrusion flow) to enable an ideal printing of PLA. As a result, there might be discrepancies among the optimal values for specific parameters between the developed filament and the selected PLA preset, which can result in a different flow behavior. Extruding too much filament, due to non-optimized flow parameters, will result in a larger line width and, thus, a larger diameter. To achieve optimal printing results, further investigations regarding other relevant software parameters must be carried out for each new formulation.

### 3.4. Solid-State Analysis Using XRD and DSC

The solid state was analyzed to determine the physical state of diclofenac sodium and other components in the matrix. Raw materials as well as the physical mixtures and 3D-printed discs of the formulations were investigated using XRD. 

**Figure 3:**
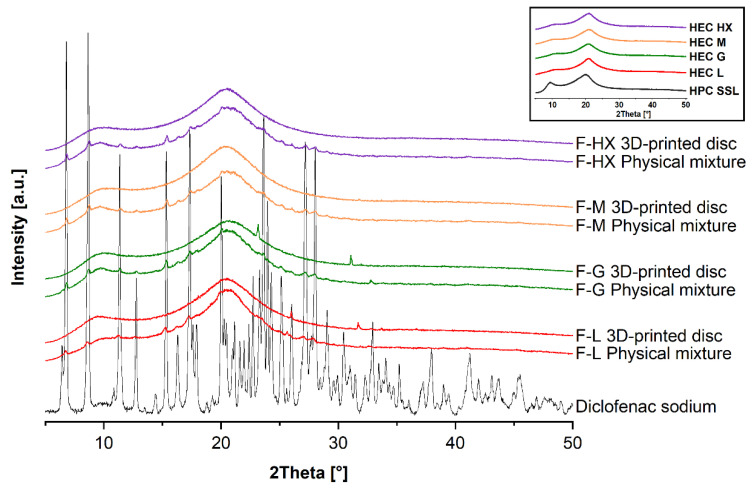
X-ray diffractograms of the raw materials (see inset), physical powder mixtures and 3D-printed formulations (F-L, F-G, F-M, F-HX).

**Figure 4:**
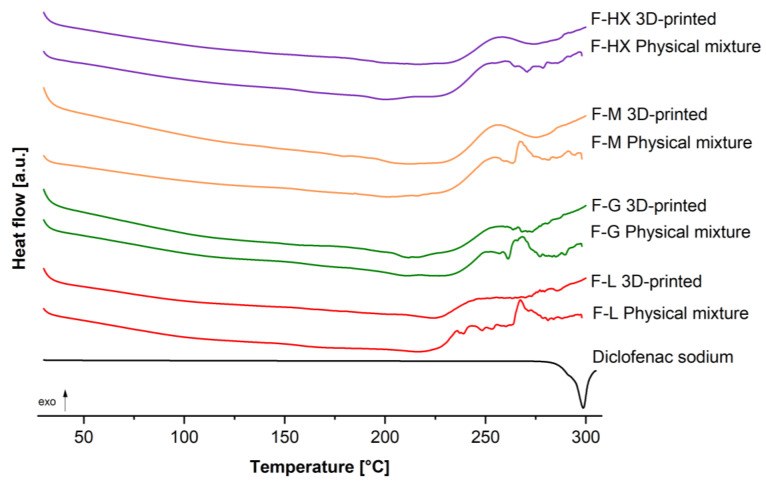
DSC thermogram of diclofenac sodium and different extrusion and 3D-printed formulations (F-L, F-G, F-M, F-HX).

Native diclofenac sodium possessed a characteristic XRD diffraction pattern with prominent peaks (2Theta (°) = 6.83, 8.68, 11.36, 15.36, 17.32, 23.64, 27.16, 28.00; Figure 3), indicating its crystalline physical form [32]. The crystalline form of diclofenac was confirmed by DSC in terms of a melting endotherm at 297 °C (Figure 4). The XRD diffraction peaks of diclofenac were also visible in each physical mixture investigated. The intensity of those peaks was lower due to the high intensity of the additional HEC and HPC amorphous halos (Figure 3, see inset) and lower relative amount of drug. Furthermore, evaluation of DSC at temperatures above 250 °C showed endothermic events, due to beginning of the thermal degradation for the polymers, as already described. Detection of distinct *T_g_* for HEC and HPC was not possible, as reported in other studies [48,49]. However, the decrease in extrusion temperatures when HPC was added to the HEC grades could indicate a decrease in *T_g_*.

For the 3D-printed discs of the formulation F-L (2Theta = 26.10°, 31.87°) and F-G (2Theta = 23.17°, 31.08°), two smaller peaks were observed in XRD, indicating that the drug might be partially crystalline. Those results were confirmed for F-G via DSC; however, the melting endotherm was very weak. These peaks could represent polymorphic forms of Diclofenac since it is known to have at least three different polymorphic forms (HD 1 and HD 2 both monoclinic forms [50] and HD 3 an orthorhombic form [51]). However, this assumption could not be clearly confirmed, so additional studies using, for example, Raman spectroscopy or hot-stage microscopy are necessary to unambiguously identify the polymorphism.

In comparison, XRD diffractograms of the F-M and F-HX discs show broad halos without additional peaks, indicating that the drug is rather amorphous, as confirmed by DSC. Those differences regarding the ability to stabilize amorphous forms can be explained with the varying MW of the used HEC polymers. As previously described [52], higher MW polymers have higher viscosity and, therefore, result in lower molecular mobility in the system, which favors the inhibition of crystallization. Due to the higher MW of HEC M (used for F-M) and HEC HX (used for F-HX), the decreased molecular mobility might stabilize the amorphous drug. 

### 3.5. Drug Content in Filaments and Drug Release of Oral Solid Dosage Forms

A uniform drug content is a decisive factor to guarantee optimal products that meet the required dosage criteria. To produce homogeneous 3D-printed dosage forms, the intermediate filaments must be investigated to ensure that they fulfill the requirement. 

The results of the drug determination in the filaments (Table 5, ‘Filament drug load’) showed slightly higher drug loads than the theoretical values (5%*w/w*). However, the relative standard deviations of the samples, taken from different parts of each filament formulation, showed little variability (≤0.46%), indicating a rather homogeneous distribution of the API. 

**Figure 5:**
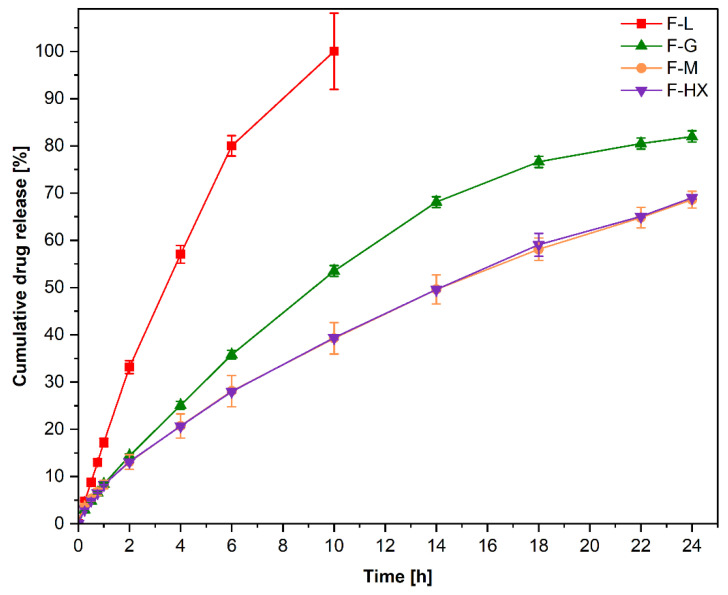
Cumulative diclofenac sodium release from 3D-printed tablets composed of different HEC/HPC combinations in phosphate buffer (pH 6.8) for 24 h. Data are presented as mean ± standard deviation (*n* = 3).

The drug release showed differences between the various formulations. We found that the F-L dosage form had the fastest release of all formulations (Figure 5). After two hours, nearly one-third (33.15 ± 1.36%) of diclofenac was released. In comparison, all other dosage forms (F-G: 14.40 ± 0.43%, F-M: 13.06 ± 1.55%, F-HX: 13.16 ± 0.21%) released less than 15% of the incorporated API within two hours. The full drug release for the F-L dosage form was achieved after 10 h, while the second fastest dosage form F-G had released 53.52 ± 1.14% of the drug at the same time. In dosage forms F-M and F-HX, which had quite similar release profiles, 39.26 ± 3.33% and 39.38 ± 0.37% of the drug was released after 10 h, respectively. The faster drug release of F-L dosage forms is attributed to the lower Mw of the HEC L grade used. The Mw, and, thus, the chain length, affect polymer mobility and polymer–solvent interactions. With increasing chain length, pronounced intermolecular entanglement occurs, which slows down the interactions between the polymer and the solvent and, therefore, reduces the dissolution rate of the polymer with a high Mw [53]. The results indicate that this effect seems to have a certain Mw threshold, where a higher Mw does not slow down the drug release further, as the F-M and F-HX drug release is very similar, although HEC HX has a higher Mw. This observation has been previously reported for the drug release of various poly(DL-lactic acid) grade tablets [54] or poly(ethylene oxide) (PEO) extrudates [55]. In the case of PEO extrudates, the researchers found that the increase in extrudate volume, upon contact with the fluid, was not substantial after exceeding a distinct Mw. This correlated with the observed drug release, which did not further decrease when the distinct Mw was reached. As we observed a swelling of the F-G, F-M and F-HX tablets, this could explain the given results and should be investigated in future studies. After 24 h of drug dissolution, the F-G dosage forms had released 81.99 ± 1.17% of diclofenac, while F-M and F-HX dosage forms had released 68.61 ± 1.78% and 69.02 ± 0.29% of the drug, respectively. The residues of these tablets were still intact after 24 h. To ensure that the unreleased drug was present in the remaining matrices, these residues were analyzed until complete dissolution. The sum of the released drug after complete dissolution of the tablet resulted in a total release of 97.00 ± 0.56% (F-HX), 98.12 ± 1.94% (F-M), and 100.00 ± 1.35% (F-G).

### 3.6. Drug Release Mechanism

The release mechanism of the released drug (*n* = 3) was evaluated, using a series of mathematical models. Therefore, release parameters and adjusted R² as statistical parameter were analyzed by DDSolver^®^ software [35]. For evaluation of the Korsmeyer-Peppas model, only the first 60% of the released drug was used [36]. 

Results revealed that all formulations achieved the highest adjusted R² for the Korsmeyer-Peppas model (Table 6). Considering the corresponding values ‘*k*’, apparent velocity, and ‘*n*’; defining the release mechanism [56] more closely, it was found that the drug release from the F-L dosage forms was the fastest, with F-G showing the lowest *k* value. Regarding the *n* values, it was noticeable that the *n* value decreased with an increasing Mw of the HEC matrix polymer, indicating a shift in the drug release mechanism. The release mechanism *n* in the Korsmeyer-Peppas model for cylindrical geometries is classified according to the observed type of behavior [56]. Since the resulting values are around 0.45 < *n* < 0.89, the underlying release mechanism is a non-Fickian release, namely an anomalous transport, which simultaneously indicates the undergoing drug diffusion and polymer relaxation/erosion. This is mainly attributed to the strong swelling and erosion behavior of the main ingredient HEC [57]. As discussed in a comparative study by Roy et al. [57], HEC showed greater water uptake than HPC, resulting in a higher degree of swelling and erosion. They also observed non-Fickian anomalous release behavior from HEC matrices [57], which is in line with our results. The fact that low-Mw formulations, such as F-L and F-G, exhibited higher *n* values, indicating a more polymer erosion-based transport, can be explained by the faster polymer erosion at lower-Mw grades [23]. Combined with the less pronounced swelling of the low-Mw formulations, particularly F-L, this leads to a faster drug release due to immediate erosion and short diffusion pathways. Consequently, the greater visually observed swelling in higher-Mw formulations leads to the development of a thick gel network and, thus, a longer diffusion path for the drug, which, in turn, causes a slower drug release [23,58].

## 4. Conclusions

FDM 3D printing enabled the production of 3D-printed controlled-release diclofenac-loaded tablets, with HEC as the main matrix polymer. While the extrusion of different grades of HEC alone was limited, ternary blends of HEC (75%*w/w*) with HPC (20%*w/w*) and diclofenac (5%*w/w*) enabled successful HME and the 3D printing of tablets, demonstrating the importance of blending so-called underused polymers with suitable excipients to facilitate processability. 

Moreover, our study supports the findings from the literature, that suitable mechanical filament properties are important to enable filament feeding during 3D printing [42]. Using a 3PB test, we were able to confirm the postulated thresholds for breaking stress and breaking distance, which allow for proper filament feeding. The F-L filament exceeded both breaking stress and breaking distance thresholds and showed good feedability in our particular printer setup (Ultimaker S5–BB 0.8 mm print core). However, the filaments that possessed values below the threshold could not be fed without a feeding support. 

The printing of the different formulations offered only neglectable differences with respect to the printing temperature. However, the subsequent drug release studies revealed differences, depending on the Mw of the polymers up to a certain threshold. Whereas an increase of the Mw of HEC from 70 kDa (HEC L) to 720 kDa (HEC M) slowed drug release, a further increase to 1000 kDa (HEC HX) resulted in no further change in drug release. The overall release mechanism was based on an anomalous transport due to drug diffusion and tablet erosion. Therefore, the study opens new possibilities for the use of HEC in combination with other excipients, to allow for different drug release profiles and to achieve drug release times ranging from 10 h (F-L) to 48 h (F-M, F-HX), making it suitable for prolonged dosing. 

Consequently, this study represents a first step to increase the knowledge about the usability of the previously underutilized HEC as a main ingredient for FDM-based 3D printing. The available Mw grades provide excellent opportunities to produce controlled-release dosage forms and customize drug release to potentially allow for personalization for the patient. However, since the feedability of the filaments was very limited, further work needs to be conducted to improve the mechanical properties (e.g., addition of plasticizer) to properly feed the filament in the FDM printer. Further studies could explore the use of HEC in FDM using more detailed analysis (e.g., rheological investigations related to printability, thermomechanical properties, tablet infill, drug loading, etc.) to gain a better understanding of its suitability for FDM 3D-printing. 

## Figures and Tables

**Table 1:** Composition of different investigated pharmaceutical formulations (F). HEC = Hydroxyethyl cellulose, HPC = Hydroxypropyl cellulose; L, G, M, HX = different Mw grades of HEC, SSL = Mw grade of HPC.

Formulation (F)	HEC L [%*w/w*]	HEC G [%*w/w*]	HEC M [%*w/w*]	HEC HX [%*w/w*]	HPC SSL [%*w/w*]	Diclofenac Sodium [%*w/w*]
F-L	75	-	-	-	20	5
F-G	-	75	-	-	20	5
F-M	-	-	75	-	20	5
F-HX	-	-	-	75	20	5

**Table 2:** Mathematical models to determine the drug release mechanism and kinetics according to Zhang et al. [35]. In the equations, F is the fraction of API released during the time t. The $k_{0},\;k_{1},\;k_{H}\;and\;k_{KP}$ values are constants for the apparent velocity of dissolution for the corresponding model, n is the diffusional exponent indicating the drug release mechanism.

Model	Equation	
Zero order	$F=k_{0}\cdot t$	(2)
First order	$F=100\cdot \left(1-e^{-k_{1}\cdot t}\right)$	(3)
Higuchi	$F=k_{H}\cdot t^{0.5}$	(4)
Korsmeyer–Peppas	$F=k_{KP}\cdot t^{n}$	(5)

**Table 3:** Hot-melt extrusion parameters of extruded HEC-HPC-Diclofenac formulations and diameter results of produced filaments.

Formulation	Extrusion Temperature [°C]	Screw Speed [rpm]	Max. Torque [Nm]	Filament Diameter [mm] Measure Points > 150
F-L	135	25	11	2.90 ± 0.04
F-G	135	25	12	2.92 ± 0.03
F-M	135	20	11	2.85 ± 0.02
F-HX	135	15	12	2.83 ± 0.02

**Table 4:** 3D-printing parameters for each formulation of 3D-printed tablets including results regarding feedability and printability of the filaments.

Formulation	Nozzle Size [mm]	Temperature [°C]		Print Speed [mm·s−1]	Layer Height [mm]	Feedability	Printability
		Nozzle	Print Bed				
F-L	0.8	200	60	25	0.3	🗸	🗸
F-G	0.8	195	60	35	0.3	✗	🗸
F-M	0.8	195	60	35	0.3	✗	🗸
F-HX	0.8	195	60	35	0.3	✗	🗸

**Table 5:** Tablet characterization results of four 3D-printed formulations (*n* = 10). Total filament drug load (*n* = 3) calculated as a percentage of filament weight.

Formulation	Tablet Weight [mg]	Tablet Diameter [mm]	Tablet Height [mm]	Filament Drug Load [%*w/w*]
F-L	358.37 ± 5.07	10.33 ± 0.04	5.01 ± 0.03	5.65 ± 0.46
F-G	354.54 ± 12.42	10.33 ± 0.06	4.97 ± 0.07	5.87 ± 0.11
F-M	360.58 ± 9.44	10.28 ± 0.04	5.00 ± 0.02	5.71 ± 0.07
F-HX	347.85 ± 11.16	10.32 ± 0.04	4.97 ± 0.02	5.45 ± 0.27

**Table 6:** Mathematical release model fitting and corresponding statistics for all formulations in phosphate buffer (pH 6.8). Note: For the Korsmeyer-Peppas model only the first 60% of the release curves (*n* = 3) was used for statistical analysis. k = constant for the apparent velocity of dissolution, n = diffusional exponent indicating the drug release mechanism.

Model	Statistic	F-L	F-G	F-M	F-HX
Zero order	adj. R²	0.938	0.926	0.942	0.945
First order	adj. R²	0.784	0.988	0.983	0.981
Higuchi	adj. R²	0.930	0.964	0.976	0.974
Korsmeyer-Peppas	adj. R²	0.996	0.999	0.999	0.999
	k	0.485	0.270	0.479	0.359
	n	0.866	0.790	0.655	0.683

## Data Availability

Not applicable.

## Supplementary Materials

The following supporting information can be downloaded at: https://www.mdpi.com/article/10.3390/pharmaceutics14102103/s1, Table S1: Characterization of different matrix polymers in terms of extrusion temperatures (°C), screw speed (rpm), extrusion torque (Nm), and filament diameter deviation (mm).
