# Supplementary material for: Evaluation of Hydroxyethyl Cellulose Grades as the Main Matrix Former to Produce 3D-Printed Controlled-Release Dosage Forms
Source: Pharmaceutics. 2022 Oct 1;14(10):2103. doi: 10.3390/pharmaceutics14102103 (PMC9609046; doi:10.3390/pharmaceutics14102103)
Supplement: Supplementary file 1 [file pharmaceutics-14-02103-s001.zip › pharmaceutics-1853943-supplementary.pdf]

Table 1. Characterization of different matrix polymers in terms of extrusion temperatures (°C), screw speed (rpm), extrusion torque (Nm) and filament diameter deviation (mm).

Polymer	Extrusion temperature [°C]	Screw speed [rpm]	Max. Torque [Nm]	Diameter deviation [mm]
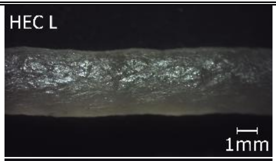	140	10	Limit reached (> 15)	> 0.05
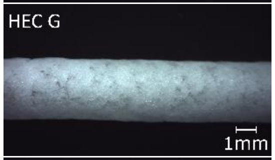	150	5	Limit reached (> 15)	> 0.05
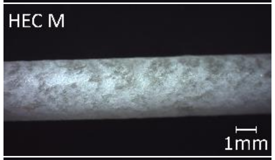	150	5	Limit reached (> 15)	> 0.05
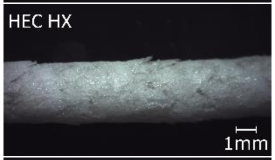	155	5	Limit reached (> 15)	> 0.05
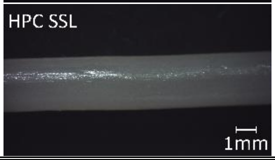	130	25	< 15	< 0.05
